# Supplementary material for: Chiral fermion reversal in chiral crystals
Source: Nat Commun. 2019 Dec 3;10:5505. doi: 10.1038/s41467-019-13435-4 (PMC6890713; doi:10.1038/s41467-019-13435-4)
Supplement: Supplementary file 1 — Supplementary Information [file 41467_2019_13435_MOESM1_ESM.pdf]

<u>Supplementary Materials</u>

Chiral fermion reversal in chiral crystals

Li *et al*.

Supplementary Note 1. Calculated bands and Fermi surfaces of RhSn with spin-orbit coupling

In the Supplementary Materials, we attach the band calculations with spin-orbit coupling (SOC) included. Supplementary Fig. 1a shows that the bands split when SOC is included compared to those without SOC in Fig. 1h. Without SOC, three bands form a threefold-degenerate point near the Fermi level (E_F) at Γ in Fig. 1h. When SOC is included, the three bands split into six spin nondegenerate bands, which form two crossing points at Γ with twofold and fourfold degeneracy, respectively (Supplementary Fig. 1b). Without SOC, four bands form a fourfold-degenerate point near E_F at R in Fig. 1h. When SOC is included, the four bands split into eight spin nondegenerate bands along Γ-R, while spin degeneracy is preserved on the Brillouin zone (BZ) boundary R-M in Supplementary Fig. 1c, which is protected by time-reversal and screw symmetries. The eight bands also form two crossing points at R with twofold and sixfold degeneracy, respectively (Supplementary Fig. 1c).

For comparison with the calculated bulk FSs without SOC in the main text, we plot the corresponding calculations with SOC in Supplementary Fig. 1, which include three-dimensional Fermi surfaces (FSs) in the bulk BZ (Supplementary Fig. 1d), projected bulk FSs on the (001) and (110) surfaces (Supplementary Fig. 1e,f), and bulk FSs in three high-symmetry planes (Supplementary Fig. 1g-i). These results show no significant differences compared with those without SOC.

Supplementary Note 2. Calculated (001) surface states of RhSn with spin-orbit coupling

In Supplementary Fig. 2, we plot the calculated (001) surface states with SOC, which show small spin splitting compared with those without SOC in Fig. 1i-k. There are four surface Fermi arcs connecting the projected FSs around $\bar{\Gamma}$ and $\bar{\mathrm{M}}$ (Supplementary Fig. 2a), and four chiral surface bands on the loops around $\bar{\Gamma}$ and $\bar{\mathrm{M}}$ (Supplementary Fig. 2b,c). The FSs around $\bar{\Gamma}$ and $\bar{\mathrm{M}}$ enclose the fourfold- and sixfold-degenerate points, respectively, indicating that they carry nonzero chiral charges ±4.

The SOC-induced band splitting in the bulk and surface states is not resolved in the experimental data. For simplicity, the discussion is conducted in the framework without SOC in the main text, as in previous studies of CoSi and RhSi.

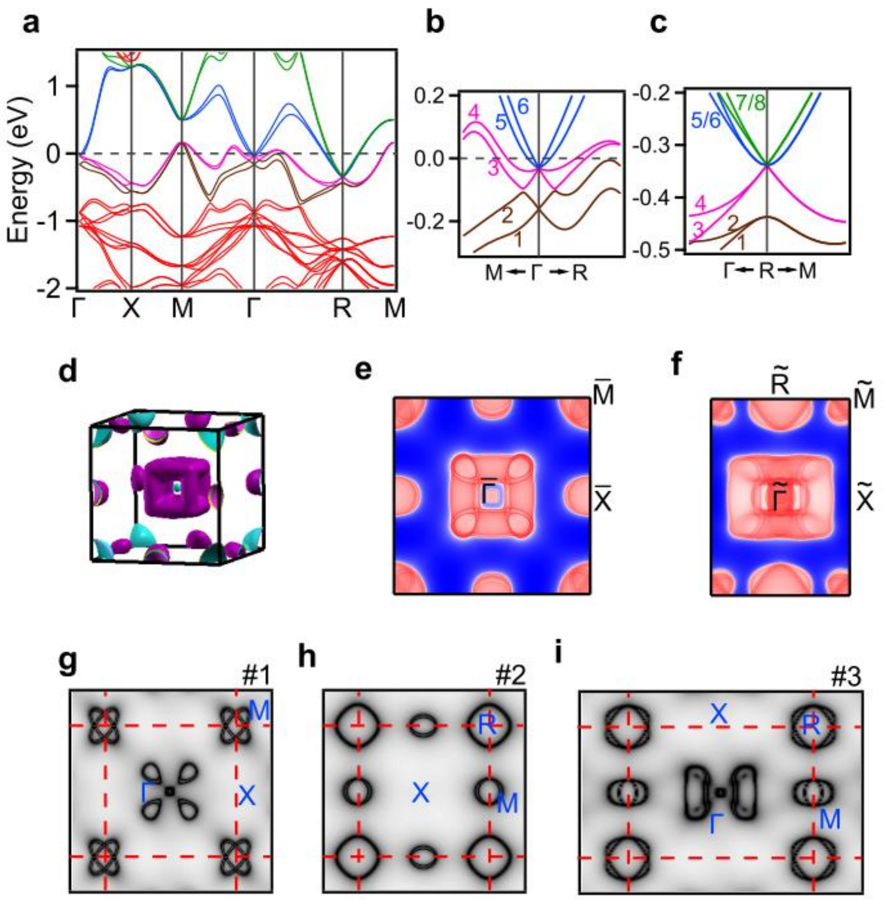

Supplementary Figure 1 | Calculated band structure and FSs of RhSn with SOC. **a**, Calculated bulk band structure of RhSn along the high-symmetry lines. **b**,**c**, Zoom-in band structures around the degenerate points at Γ and R, respectively. **d**, Calculated FSs of RhSn in the bulk BZ. **e**,**f**, Projected bulk FSs on the (001) and (110) surfaces, respectively. **g**,**h**,**i**, Calculated bulk FSs in three high-symmetry planes #1, #2 and #3, whose locations in the bulk BZ are indicated in Fig. 2g.

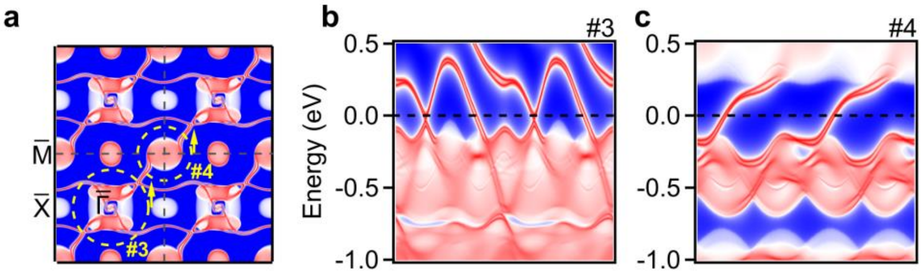

Supplementary Figure 2 | Calculated surface states of RhSn with SOC. a, (001)-surface states of RhSn. **b**,**c**, Surface band structures on loops #3 and #4, respectively.
